# Nanotubes enable intercellular communication in early-branching eukaryotes

**DOI:** 10.1093/pnasnexus/pgag238

**Published:** 2026-07-28

**Authors:** Harikumar R Suma, Robert R Kay, Sandeep M Eswarappa, Pierre Stallforth

**Affiliations:** Department of Paleobiotechnology, Leibniz Institute for Natural Product Research and Infection Biology—Leibniz-HKI, Beutenbergstrasse 11a, 07745 Jena, Thuringia, Germany; Cluster of Excellence Balance of the Microverse, Friedrich Schiller University, Fürstengraben 1, 07743 Jena, Thuringia, Germany; Laboratory of Molecular Biology, Medical Research Council, Francis Crick Avenue, Trumpington, Cambridge CB2 0QH, United Kingdom; Department of Biochemistry, Indian Institute of Science, Bengaluru 560012, Karnataka, India; Department of Paleobiotechnology, Leibniz Institute for Natural Product Research and Infection Biology—Leibniz-HKI, Beutenbergstrasse 11a, 07745 Jena, Thuringia, Germany; Faculty of Chemistry and Earth Sciences, Friedrich Schiller University (FSU), Humboldtstrasse 10, 07743 Jena, Thuringia, Germany

**Keywords:** multicellularity, tunneling nanotube, actin, amoebae, *Dictyostelium discoideum*, membrane continuity, supercellularity

## Abstract

Social amoebae, despite being unicellular organisms, exhibit multicellular phenotypes that require well-tuned inter- and intracellular communication modes. The social amoeba *Dictyostelium discoideum* is an excellent model to study multicellularity and metazoan cell signaling. While communication based on secreted small molecules has been described, juxtacrine connections were so far an unexplored intercellular communication mechanism in amoebae. Using fluorescence labeling and confocal microscopy, we identified de novo formation of tunneling nanotubes (TNTs) in amoebae. Live cell imaging revealed that these nanotubes are actin-rich protrusions that facilitate cargo transport by enabling membrane continuity between the connected cells. The identification of nanotubes in different amoebal species highlights the relevance of these protrusions within natural microbial communities since they act as a complementary mechanism for the transfer of essential cellular cargo. Remarkably, TNT-mediated connectivity persists under conditions that perturb cytoskeletal dynamics, indicating that nanotube-based communication is robust and adaptable. TNTs represent a novel juxtacrine communication machinery that promotes cell-to-cell communication and coordination in early eukaryotes and could play a key role in the emergence of multicellularity.

Significance statement Tunneling nanotubes (TNTs) are actin-supported membrane protrusions that enable the exchange of diverse cellular cargo in prokaryotes and higher eukaryotes. However, their occurrence and functional relevance in early-branching eukaryotes have not been clearly established. Here, we demonstrate that social amoebae form de novo TNTs that function as a juxtacrine communication route and support intercellular transport of cytoplasmic components and organelles. TNTs represent a novel juxtacrine communication machinery that promotes cell-to-cell communication and coordination in early eukaryotes and could play a key role in the emergence of multicellularity. Our findings also position amoebae as a suitable model to study TNT biology and suggest that TNT-like supracellular networks represent an evolutionarily conserved mechanism for long-distance intercellular communication.

## Introduction

Cell-to-cell communication is a prerequisite for establishing and maintaining cellular homeostasis, whether within unicellular microbial communities or in multicellular organisms (1, 2). Chemical communication, widely considered the classical form of cell-to-cell communication, occurs in a paracrine manner through secreted small molecules or via extracellular vesicles (3, 4). Such communication machinery is highly effective for long-distance communication, as some signals can travel long distances to stimulate distant cells.

On the other hand, contact-based juxtacrine communication occurs through intercellular protrusions that establish direct physical connections between cells. Plasmodesmata in plant cells (5), gap junctions in animal cells (6), and septal pores in fungi (7) are some examples of contact-based communication structures that connect cells in close proximity and mediate the exchange of various biological signals. Even in prokaryotes, exchange of information can occur through physical interactions between neighboring cells (8). One such example is bacterial conjugation, in which DNA is transferred between cells through a tube-like structure known as a pilus (9).

Occasionally, long-distance cell-to-cell communication is made possible through contact-based intercellular protrusions known as tunneling nanotubes (TNTs) (10). Unlike other juxtacrine communication structures such as plasmodesmata (50–200 nm), fungal septal pores (100–500 nm), bacterial pili (0.5–5 µm), and gap junctions in animal cells (2–4 nm), which are restricted to connecting adjacent cells, tunneling nanotubes are long membrane protrusions that extend to distances over 100 μm, supported by cytoskeletal elements, particularly F-actin filaments (5, 6, 9–15). Initially reported in metazoans, TNTs allow for uni- and bidirectional transfer of genetic materials, proteins, organelles, and other small molecules through passive and active transport mechanisms (16–19). Because of this feature, their relevance has been implicated in several pathophysiological conditions and disease transmission (20–24).

Beyond the metazoan phyla, analogs of nanotubes have been reported in various other life forms, highlighting the significance of TNT-based supercellularity in cell-to-cell communication (25–28). The existence of open-ended membrane channels, in particular TNTs, is suggested to be the fundamental principle of supercellularity, i.e. multicellularity beyond the traditional cellular assemblies (15). As TNT formation enables membrane continuity across different scales—from bacteria to eukaryotic tissues—these structures serve as supracellular assemblies crucial for cellular communication and maintaining cellular homeostasis.

Among the nonmammalian model systems, amoebae are a particular group of eukaryotes as they exhibit morphogenesis between a unicellular vegetative stage and a multicellular development stage during their life cycle (29, 30). Social amoebae, including *Dictyostelium discoideum* and *Polysphondylium violaceum*, are used as model systems to understand different aspects of cell biology (31, 32) and host–pathogen interactions (33–35), and chemotaxis (36, 37). *Dictyostelium discoideum* is also used to study the underlying principles of several metazoan biological processes at the level of a whole organism, which is still difficult to achieve using mammalian cell culture-based systems (38–41). Similar to other eukaryotes, amoebae also employ different intercellular mechanisms for cell-to-cell communication during their uni- and multicellular life stages. These mechanisms mainly occur in a paracrine manner, mediated through chemical messengers such as cAMP, dictyodene, and differentiation-inducing factor (DIF) (29, 42–44). The functional analogy of such processes between amoebae and other amoebocytes, including macrophages, underscores the importance of investigating how these cells communicate and coordinate.

Moreover, beyond the metazoan phyla, the presence of contact-based communication mechanisms such as TNTs in early-branching eukaryotes are not well understood. Furthermore, the signals leading to the formation of TNTs and their functional significance remain poorly characterized. Here, we have examined the relevance of TNTs in amoebae, an early-branching eukaryote (45), by equipping *D. discoideum* cells with fluorescent labels for cytoskeletal elements and other organelles. Intriguingly, our imaging studies reveal that de novo TNTs act as a juxtacrine mode of communication in amoebae. They function as conduits for intercellular transport under both normal and cytoskeleton-perturbed conditions, further establishing the functional significance of TNTs in amoebae. These results emphasize the importance of TNT-based communication in amoebae. The distinct structural and functional analogy of nanotube-based communication found throughout eukaryotes, including amoebae, down to prokaryotes, highlights a universal, conserved, and highly regulated intercellular communication pathway.

## Results

### Actin-based nanotubes connect amoebae cells

**Figure 1:**
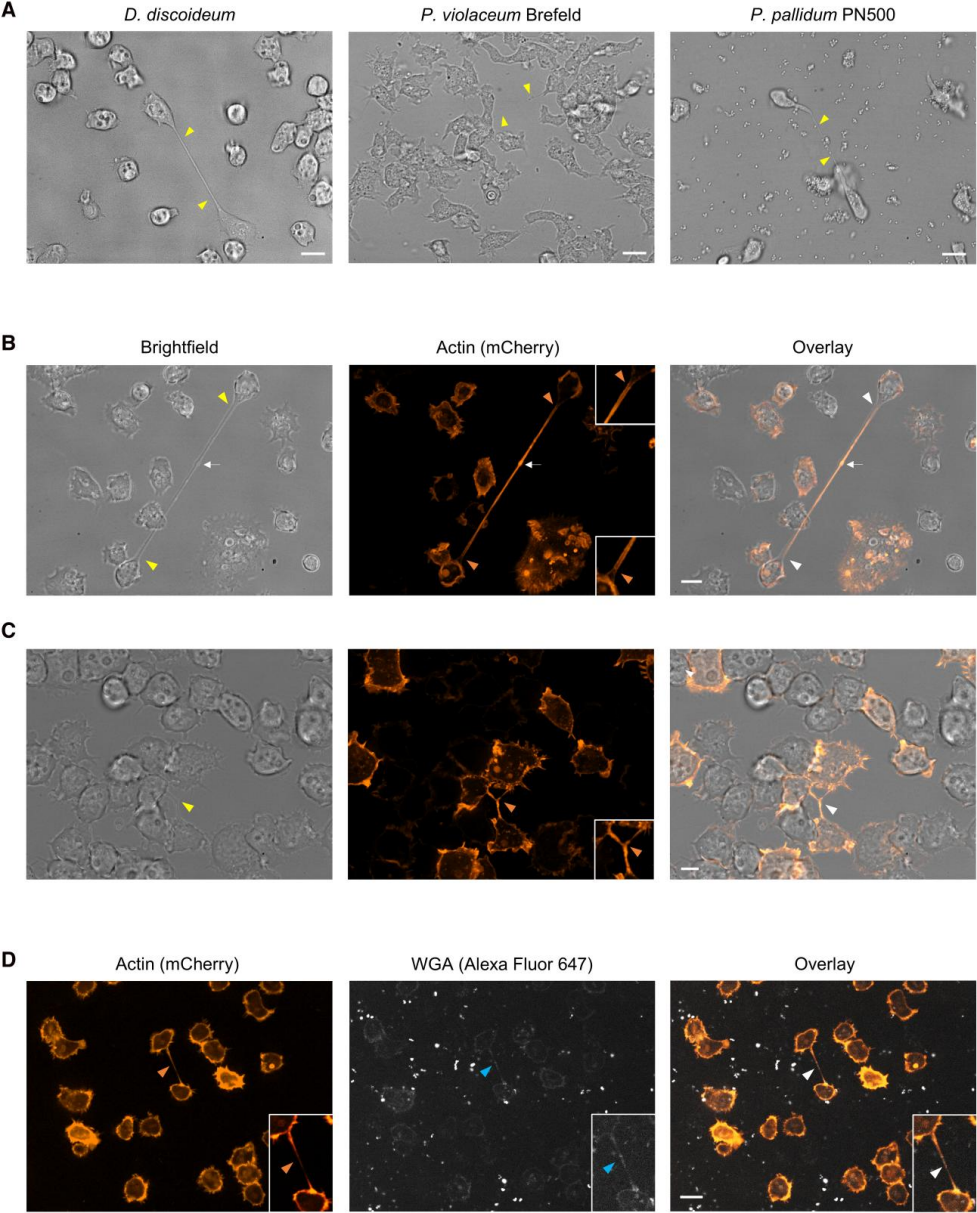
Identification of TNTs in amoebae. A) Brightfield images of various amoebal species showing the formation of TNT-like protrusions between cells. Yellow arrowheads indicate the intercellular protrusions across different species. Scale bars, 10 µm. B, C) Representative maximum intensity projection images showing the architecture of TNTs formed between *D. discoideum* VGA4-LAC cells. Images of B) single (ca. 87 μm) and C) branched TNTs connecting multiple *Dictyostelium* cells. The nanotube structures were labeled positive for F-actin, and the inset images show enlarged depictions of TNTs. D) *Dictyostelium discoideum* VGA4-LAC cells stained with WGA Alexa Fluor 647 conjugate indicate that TNTs enable membrane continuity between connected cells. Blue arrowheads indicate WGA staining of the nanotube. Yellow, orange, and white arrowheads indicate the TNTs in brightfield, actin-positive, and overlay with brightfield, respectively. White arrows indicate the presence of a gondola on the TNT. Scale bars, 10 µm.

To investigate the formation and function of TNTs in early eukaryotes, we examined whether amoebae are able to form contact-based intercellular connections using a microscopy-based approach. Using brightfield imaging, we observed nanotube-like membrane protrusions connecting cell pairs within populations of various social amoebae, including *D. discoideum*, *P. violaceum*, and *Polysphondylium pallidum* PN500 (Fig. 1A). Intriguingly, in rare instances, such protrusions were also observed connecting two different species of amoebae, *D. discoideum* and *P. violaceum* (Fig. S1A and B). To our knowledge, this is the first documented occurrence of such membrane protrusions in amoebae. So, to further characterize these protrusions, we introduced the *LifeAct-mCherry* (LAC) *mRuby3–α-tubulin* (RT) cassettes into the *D. discoideum* VGA4 strain already expressing the VatM-GFP fusion protein (see Materials and methods).

Live-cell imaging of *D. discoideum* VGA4-LAC and VGA4-RT strains revealed that these tubular structures were fluorescently labeled by the LifeAct peptide. Hence, the localization of F-actin filaments within these structures was confirmed (Fig. 1B and C). Incidentally, these protrusions showed heterogeneity in their morphology and dimensions, as certain nanotubular connections also appeared to have a branched morphology connecting more than two amoebae cells (Fig. 1C). The lengths of these structures ranged from 10 to 90 μm with a width of 0.5 to 1.2 μm (Fig. 1B and C). The longest nanotubular structure we observed was over 400 μm (Fig. S1C). Additionally, these protrusions enabled membrane continuity (Fig. 1D) and did not appear to be remnants of incomplete cytokinesis (Fig. S1D), highlighting that these nanotubular structures are formed de novo. These structures were dynamic and did not adhere to the substratum (Figs. S2 and S3). Taken together, our data strongly suggest that these structures formed among amoebae met the primary criteria to be classified as TNTs (10, 46, 47). Therefore, we could show that TNT formation occurs across multiple species of amoebae and enables membrane continuity between the connected cells.

### Amoebal TNTs mediate the transport of organelles

To determine whether TNTs can facilitate cargo transfer, we used the *D. discoideum* VGA4-LAC strain coexpressing VatM-GFP and LAC fusion proteins. The *vatM* gene encodes for the transmembrane subunit of the vacuolar H^+^-ATPase (V-ATPase) enzyme, which is responsible for the acidification of various endomembrane organelles through its localization to their membranes (48). In *Dictyostelium*, VatM-GFP fusion protein localizes to the membranes of the endocytic and endolysosomal vesicles, as well as to the contractile vacuole complex (49–51). Additionally, to visualize the transfer of mitochondria and peroxisomes, we stained amoebae using the BioTracker 408 Mitochondria and Peroxi_SPY™555 dyes, respectively.

**Figure 2:**
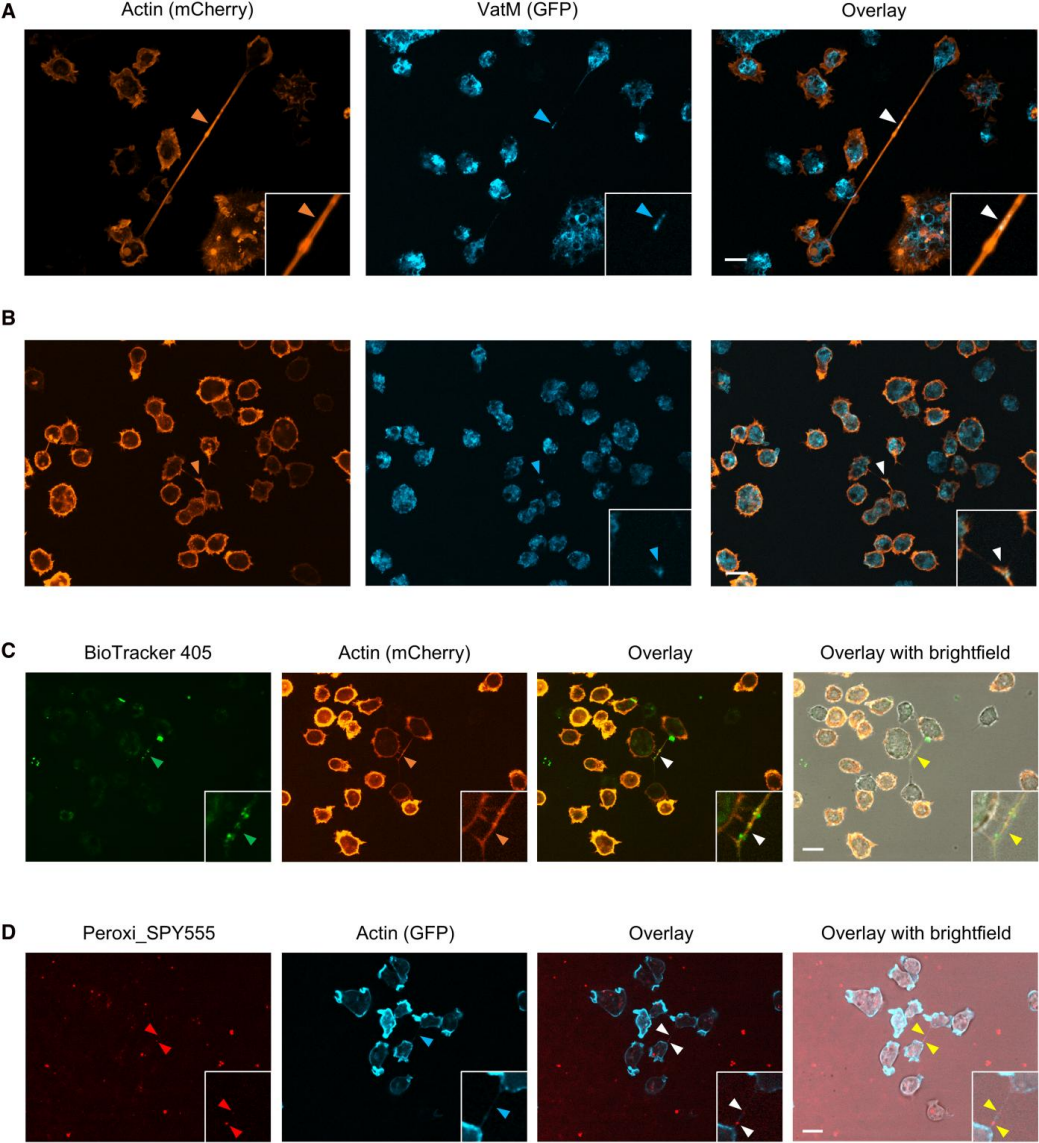
Intercellular transfer of organelles between TNT-connected cells. A, B) The transport of endomembrane organelles (blue) between actin-labeled (orange) amoebae. Organelles could be detected inside A) a single, B) branched TNTs connecting amoebae. Orange arrowheads indicate actin-positive TNTs, blue arrowheads indicate organelles, and white arrowheads indicate the overlay of organelles within these TNTs. Scale bars, 10 µm. C, D) TNTs between *D. discoideum* VGA4-LAC cells containing signals for other punctate organelles. The transfer of C) mitochondria and D) peroxisomes is indicated by the distribution of the dyes, BioTracker 408 Mitochondria and Peroxi_SPY™555, respectively, in the nanotubes. Orange arrowheads indicate actin-positive TNTs, green and red arrowheads indicate mitochondria and peroxisomes, respectively. White and yellow arrowheads indicate the overlay of organelles within these TNTs. Scale bars, 10 µm.

During imaging, we detected the presence of membrane-bound organelles inside amoebal TNTs. They were detected within single (Fig. 2A) and branched TNTs (Fig. 2B). Intriguingly, TNTs were positively stained for mitochondria and peroxisomes, also indicating that the transport of other organelles is also possible through amoebal TNTs (Fig. 2C and D). The localization of microtubules was not detected inside the amoebal TNTs (Fig. S4A and B). Additionally, it was observed that distension on the nanotubes resulted in the formation of gondola structures—a *bona fide* feature of TNTs (13, 52) (Figs. 1B and S4C and D). In certain cases, signals for organelles could be detected inside these gondolas, indicative of their function as a transport vehicle (bottom panel; Fig. S4C). Furthermore, it was also possible to detect actin-positive TNTs exhibiting transfer of fluorescent organelles (Fig. S5). These findings suggest the role of TNTs as a mechanism for intercellular transport between proximal and distant cells, further highlighting the functional significance of nanotubes in the cell-to-cell communication of amoebae.

### Effect of actin-depolymerizing agents on amoebal TNT formation

Since TNTs are primarily supported by the F-actin cytoskeleton, actin-depolymerizing agents such as latrunculin B (lat B) have been shown to predominantly inhibit the formation of TNTs in various mammalian cells (10, 53, 54). However, some studies have shown that lat B treatment does not inhibit nanotube formation and, in one instance, even an upregulation has been observed (52, 55, 56). Additionally, lat B treatment of immune cells exhibiting migratory behavior resulted in the rapid upregulation of intraspecific and interspecific TNTs with cancer cells (57). Hence, to explore the role of actin depolymerization in amoebal TNT formation, we induced cytoskeletal stress (58, 59) in *D. discoideum* AX2 cells expressing LifeAct-GFP (LAG) using three actin-depolymerizing agents—latrunculin B (lat B), cytochalasin D (cyt D), and latrunculin A (lat A), which have previously shown to alter the actin cytoskeleton dynamics in *Dictyostelium* cells (60, 61).

**Figure 3:**
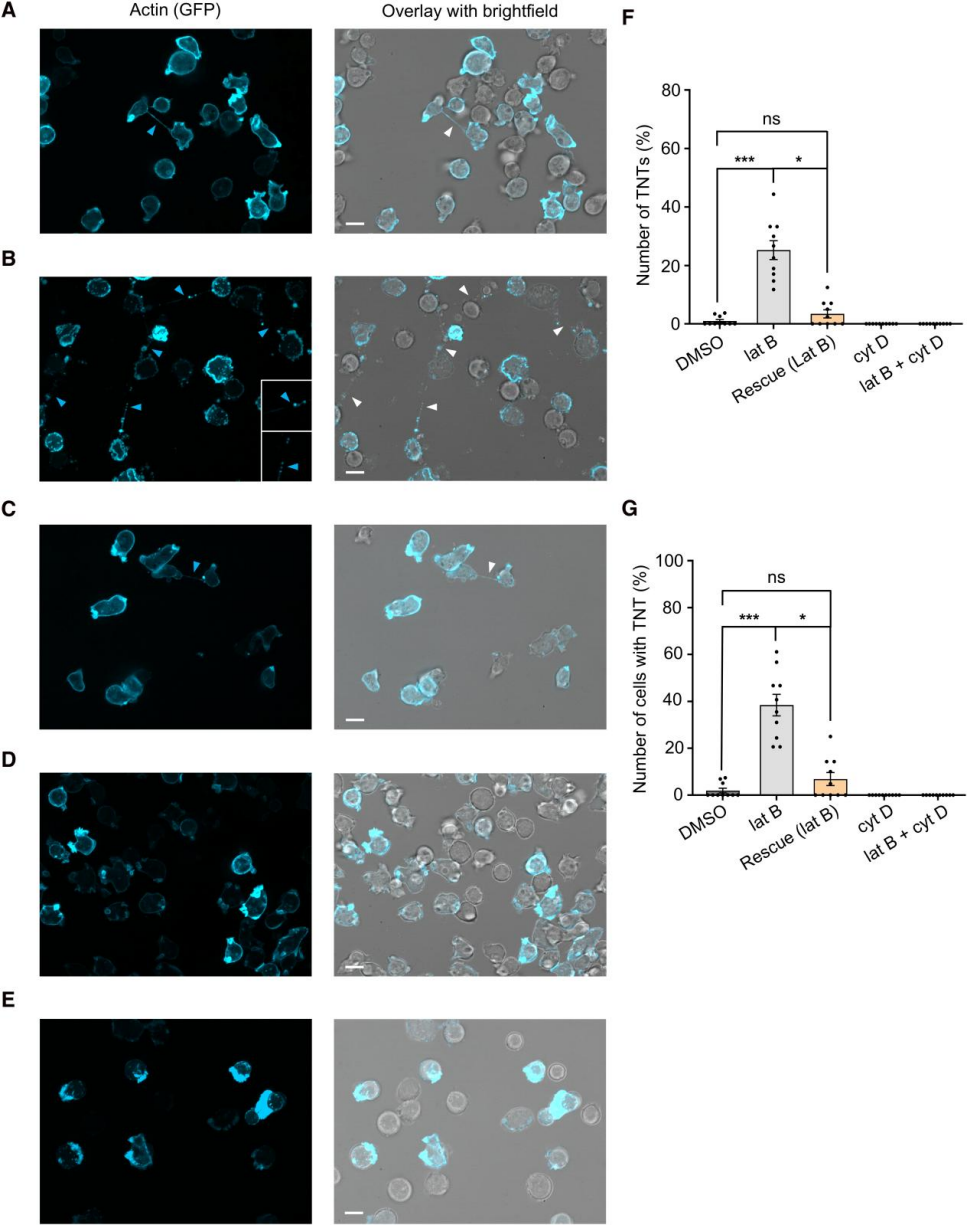
Differences in TNT formation during drug treatments. A) Amoebae exhibit their characteristic amoeboid morphology, and the formation of TNTs happens in control cells treated with DMSO. B) Effect of lat B treatment (10 μM) on TNT formation in amoebae. An increase in the number of TNTs could be observed in lat B-treated cells. Actin aggregates were also detected within TNTs (B insets). C) Recovery of cells after lat B treatment. Cells rescued from lat B treatment returned to their normal amoeboid morphology, and there was a decline in TNTs. D) Effect of cytochalasin D treatment (50 μM) on TNT formation in amoebae. No TNTs could be detected in cells treated with cyt D. E) Effect of combinatorial drug treatment (lat B and cyt D) on TNT formation. No TNTs could be detected between the cells. White arrowheads indicate the GFP-positive TNTs. Scale bars, 10 µm. F) Quantification of TNTs formed between cells. G) Comparison of the percentages of cells connected by TNTs. Both (E) and (G) were performed in three independent experiments. Data shown are mean ± SE (*n* = 10 images). Graphs are representative of three independent experiments. Statistical significance was determined using Kruskal–Wallis test followed by Dunn's *post hoc* test for multiple comparisons (symbols: ns, not significant, *P < 0.05, and ***P < 0.001). F) H(4, N = 50) = 36.50, *P* < 0.0001), G) H(4, N = 50) = 36.04, *P* < 0.0001.

When compared to the control cells, the treatment with lat B (10 μM) led to the disassembly of F-actin structures as the cells lost their characteristic amoeboid movement, forcing them to adopt an aberrant morphology with reduced motility (Fig. 3A and B). This phenomenon can be retraced to decreased cortical tension, which is a direct consequence of disruption of the actin-based cytoskeleton (62). However, there was a significant increase in the number of TNTs formed among the lat B-treated cells when compared to the control cells (*P* < 0.001; Fig. 3A and B). While membrane continuity was still maintained (Fig. S6A), F-actin filaments were largely replaced by actin aggregates (63), which were heterogeneously distributed along partially and fully formed TNTs, instead of being uniformly distributed along their length (Fig. 3B insets, Fig. S6B).

Interestingly, the cells rescued from lat B treatment regained their characteristic amoeboid morphology (Fig. 3C). However, TNTs were not detected during the cyt D treatment (50 μM) and the cotreatment of lat B and cyt D (Fig. 3D–G). The lat B-treated cells (25.3 ± 3.2%; Fig. 3B) showed a ∼25-fold increase in TNTs compared to the control cells (0.98 ± 0.5%; Fig. 3F). This increase was also observed in the number of cells connected with TNTs (38.4 ± 4.5% for lat B-treated and 1.94 ± 1% for control; Fig. 3G). However, a significant decrease (*P* < 0.05) in the number of TNTs (3.4 ± 1.4%) was observed in cells rescued from lat B treatment when compared to the lat B-treated cells (25.3 ± 3.2%; Fig. 3F).

Furthermore, we treated the amoebae cells with lat A (5 μM), a structural analog of lat B (64). Because of its higher binding affinity and stability, lat A is generally regarded as more potent in disrupting actin polymerization than lat B (65). Treatments with lat A and lat B-induced membrane pearling, which is considered the final stage of actin depolymerization (66). Intriguingly, TNT formation was also observed during lat A treatment (Fig. S6C). Hence, we could demonstrate that cytoskeletal stress induced by latrunculins can be one of the stimuli for TNT biogenesis, and this process is reversible in amoebae. Furthermore, these nanotubular structures remain stable even after the disassembly of actin filaments.

### Characterization of TNT formation under actin-disrupting conditions in amoebae

Because TNT formation was observed in amoebae following the disruption of actin cytoskeleton, we hypothesized that other cytoskeletal elements, such as microtubules, may compensate and stabilize the nanotubes. To investigate this possibility, we co-expressed fluorescent markers for actin filaments (LAG) and microtubules (RT) in *D. discoideum* AX2 cells (hereafter *D. discoideum* LAG-RT). Cytoskeletal stress was induced by the exposure to either lat B alone or a co-treatment with lat B and nocodazole (noc), a microtubule depolymerization agent (61, 67).

**Figure 4:**
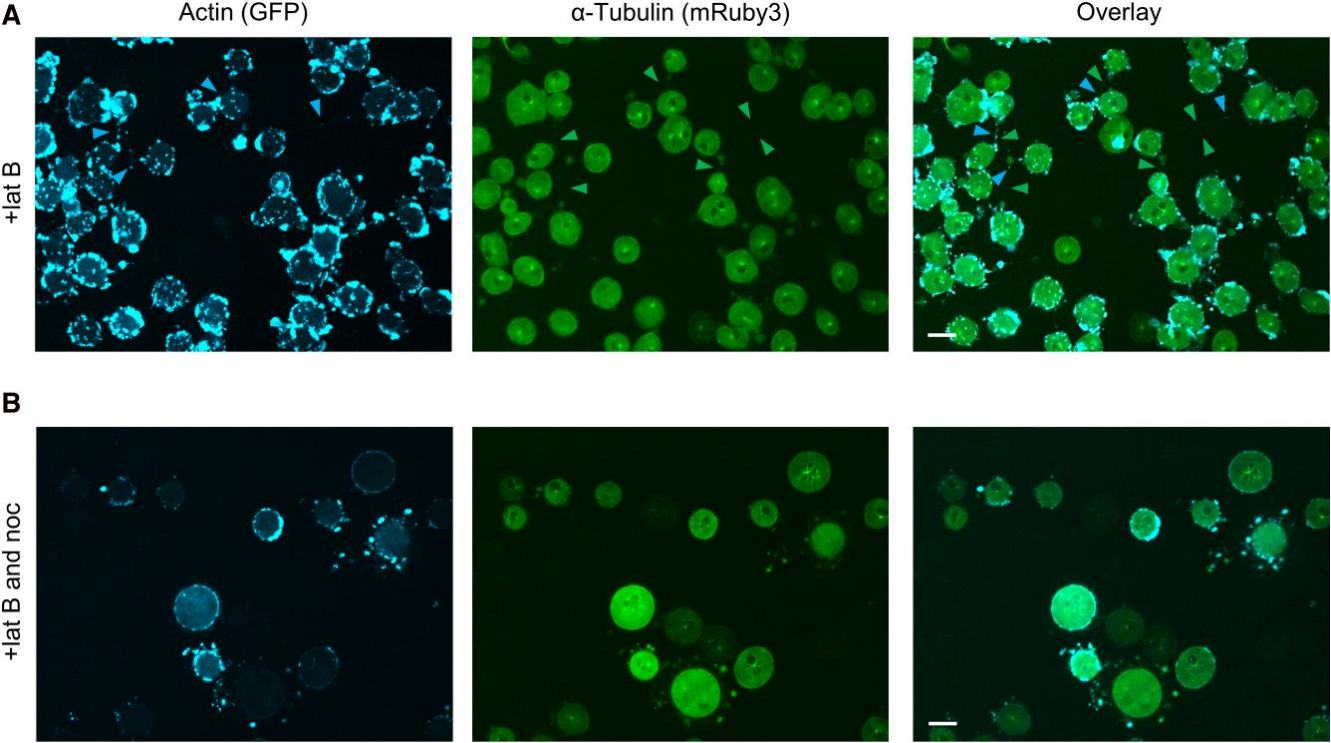
Cytoskeletal remodeling inside TNTs following actin cytoskeleton disruption. A) Lat B treatment (10 μM) of *D. discoideum* LAG-RT cells showed the presence of microtubules (green) inside TNTs, along with actin aggregates (blue). B) The co-treatment of lat B (10 μM) and noc (66 μM) led to the complete absence of TNTs as both actin cytoskeleton and microtubule filaments were disrupted. Scale bars, 10 µm.

Intriguingly, lat B-treated *D. discoideum* LAG-RT cells displayed a faint yet uniform signal for microtubules within the nanotubes, along with actin aggregates (Fig. 4A). However, under normal conditions, microtubules were not detected within amoebal nanotubes (Fig. S4A and B). In contrast, co-treatment of lat B and noc resulted in the disruption of both actin cytoskeleton and microtubule filaments, resulting in the complete absence of TNTs (Fig. 4B). These findings strongly suggest that even when the actin cytoskeleton is disrupted, microtubules may adopt a compensatory role in maintaining the structural integrity to nanotubes. Overall, these observations highlight that cytoskeletal remodeling in response to cytoskeletal stress could enable amoebae to maintain TNT architecture.

**Figure 5:**
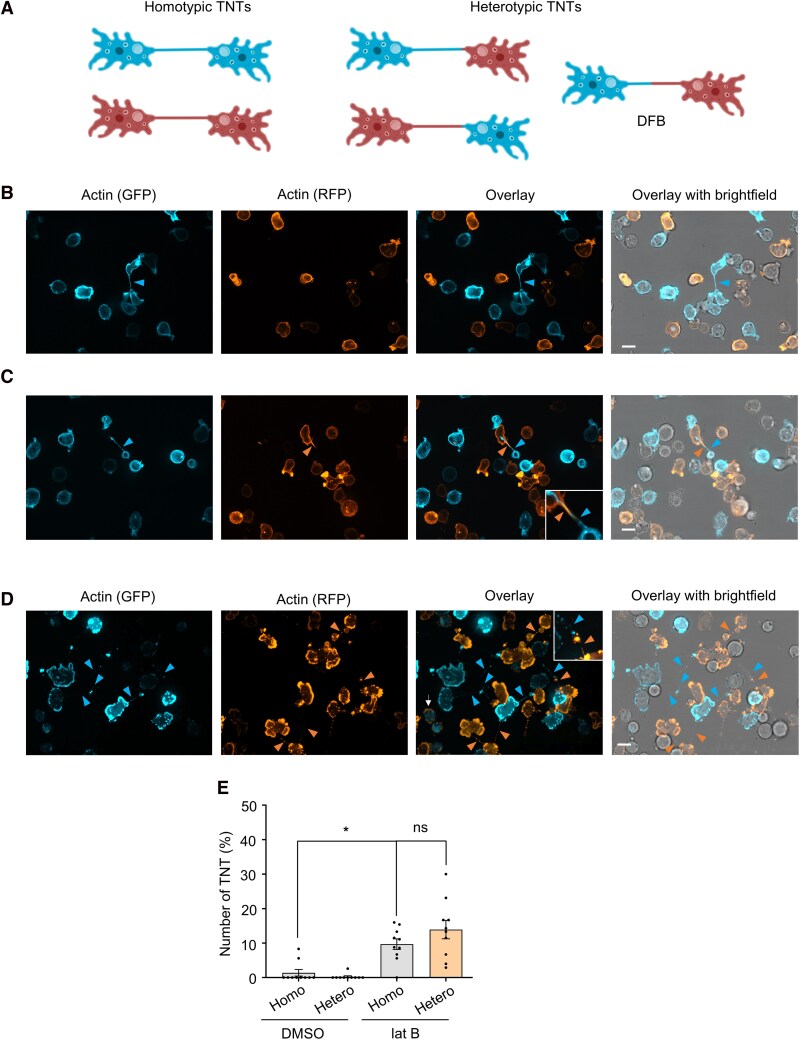
Stress-induced biogenesis of TNTs enables the exchange of essential cargo. A) Schematic of homotypic and heterotypic TNTs along with a DFBs—a type of heterotypic TNT (72), discussed in this study. DFBs were also identified in the amoebal co-culture. B) A homotypic TNT was detected in the co-culture of *D. discoideum* cells LAG and LAR cells. C) Heterotypic TNT could also be detected in the co-culture under control conditions. D) Effect of latrunculin B treatment (10 μM) on the co-culture of amoebae. Formation of homotypic and heterotypic TNTs was detected between the isogenic strains of *D. discoideum* AX2. Blue and orange arrowheads indicate the GFP- and RFP-positive TNTs, respectively. Inset images in C and D show DFBs between amoebae. White arrow indicates the localization of actin aggregates (orange) surrounding the LAG cells (similar to Fig. 6A), highlighting the transfer of actin between amoebae. Scale bar, 10 µm. E) Comparison of the types of TNTs formed between control and lat B-treated cells. Data shown are mean ± SE (*n* = 10 images). Graphs are representative of three independent experiments. Statistical significance was determined using Kruskal–Wallis test (H(3, N = 40) = 27.25, *P* < 0.0001) followed by Dunn's *post hoc* test for multiple comparisons (symbols: ns, not significant, *P < 0.05). The illustrations were created with BioRender.com.

TNTs have been described to form between heterogeneous cell populations and enable organelle trafficking (24, 57, 68–71). We thus performed a co-culture between isogenic strains of *D. discoideum* LAG and LifeAct-RFP (LAR) fusion proteins. Lat B treatment was used to induce cytoskeletal stress in the co-cultures. To clearly distinguish the TNT variants in the co-cultures, we used the following criteria: homotypic are those TNTs formed only among GFP- or RFP-positive cells (Fig. 5A and B), whereas heterotypic TNTs are formed between GFP- and RFP-positive cells (Fig. 5A–C).

In the co-culture subjected to lat B treatment, there was a significant increase in both homotypic (9.7 ± 1.5%) and heterotypic (13.9 ± 2.6%) TNTs (Fig. 5D and E). Even though the addition of lat B led to an overall increase in the number of TNTs, the differences among the number of homotypic and heterotypic TNTs were comparable (*P* > 0.9; Fig. 4E). We could also observe filopodial extensions from both GFP- and RFP-positive cells reaching out to form TNTs (Fig. 5C and D insets). These structures are similar to double filopodial bridges (DFB), previously reported in mammalian cells (72). In the control cells, we could detect a few homotypic (1.4 ± 0.9%) and even fewer heterotypic (0.26 ± 0.2%) TNTs. This suggests that under normal conditions, TNT formation in amoebae could be a rare phenomenon that could account for the lack of previous reports of TNTs in these organisms.

**Figure 6:**
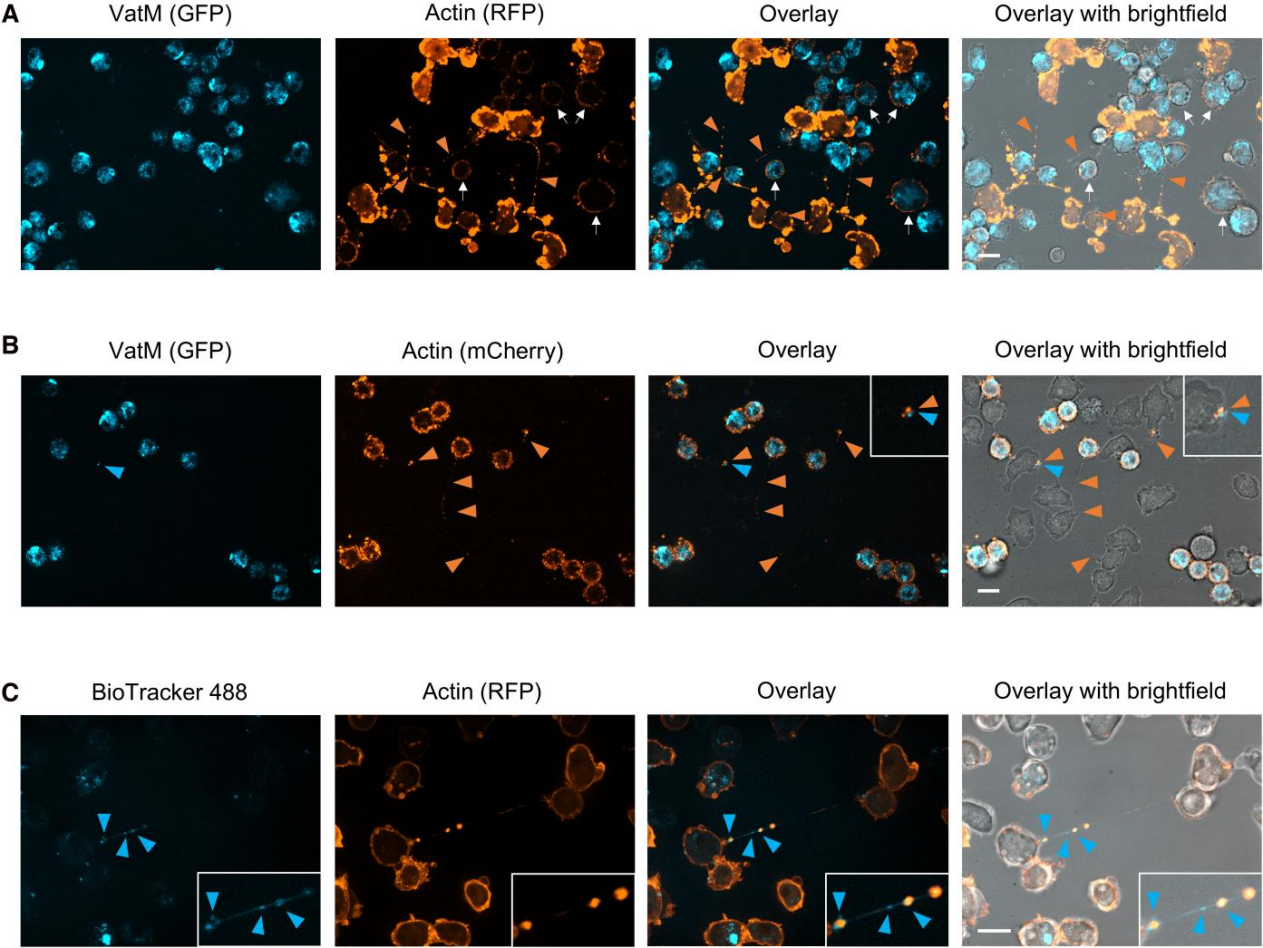
Cytoskeletal perturbation promotes TNT formation and enables cargo transport. A) Heterotypic TNTs are formed during lat B treatment of the co-culture *D. discoideum* VGA4 cells and *D. discoideum* LAR cells. These TNTs enable the transfer of actin aggregates from LAR cells to nonactin-labeled VGA4 cells. White arrows indicate the actin aggregates (orange) surrounding the VGA4 cells, highlighting the transfer of actin between amoebae. Scale bar, 10 µm. B) Lat B treatment of co-culture between unlabeled *D. discoideum* AX2 cells and *D. discoideum* VGA4-LAC cells. Heterotypic TNTs were detected between the labeled and unlabeled cells after lat B treatment. Actin aggregates (orange) and organelles (blue) were bound to unlabeled cells connected to TNTs from fluorescently labeled amoebae (inset). Blue and orange arrowheads indicate the organelle distribution and actin-positive TNTs, respectively. Scale bar, 10 µm. C) Lat B treatment of *D. discoideum* LAR cells, later stained by BioTracker 488 Mitochondria Dye. The distribution of mitochondrial particles inside the amoebal TNTs indicates the transfer of mitochondria through the TNTs (inset). Multiple gondolas positively stained for mitochondria could also be seen on the nanotube. Blue and orange arrowheads indicate the signal for mitochondria and actin-positive TNTs, respectively. Scale bar, 10 µm.

Notably, the visualization of RFP-positive actin aggregates surrounding nonactin-labeled *D. discoideum* VGA4 cells connected by fluorescent TNTs during lat B treatment suggests the transfer of actin aggregates between amoebae (Fig. 6A). Further highlighting that cytoskeletal stress can induce the formation of heterotypic TNTs in amoebae through intercellular protrusions that extend to neighboring cells, irrespective of the strains (Fig. S6B).

To further investigate the delivery of cargo during cytoskeletal stress, we investigated the transfer of organelles by setting up a co-culture experiment involving unlabeled *D. discoideum* AX2 and *D. discoideum* VGA4-LAC cells. In this co-culture, heterotypic TNTs were identified between both strains. At the same time, signals for actin aggregates and organelles were detected to be bound with the unlabeled cells connected by fluorescent TNTs (Fig. 6B). Further, we performed mitochondrial staining of *D. discoideum* LAR cells that were subjected to lat B treatment. We visualized TNTs stained positively for mitochondria inside a gondola (Fig. 6C), suggesting mitochondrial trafficking occurs during cytoskeletal stress conditions. Overall, our data reveal a complementary role for TNTs in intercellular communication, enabling amoebae to transfer essential cargo during cytoskeletal stress.

## Discussion

Evolving a wide range of paracrine cell-to-cell communication mechanisms has enabled Amoebozoa to adapt to various environmental conditions. Interestingly, our results reveal the formation of TNTs—a juxtacrine communication machinery—across multiple species within this lower eukaryotic phylum, generally regarded as a well-studied nonmammalian model system.

The presence of F-actin filaments along the length of amoebal TNTs is a feature that is synonymous with mammalian TNTs (Fig. 1). Even though less studied, prokaryotic nanotubes are likely composed of lipid membranes without internal cytoskeletal elements (73). Compared to their prokaryotic counterparts, the dimensions of amoebal TNTs closely resemble those of mammalian TNTs. The branched morphology is often an atypical form of TNTs architecture, although similar structures have been reported in prokaryotes and mammalian cells, including macrophages (17, 25, 74). The presence of such structural heterogeneity in amoebal TNTs may enable multicell connectivity and highlight the functional significance of TNT-based supercellularity in cell-to-cell communication.

Several models have been proposed to explain how TNTs are formed, including (i) filopodial extension, in which actin-rich protrusions extend toward neighboring cells and (ii) cell dislodgment, in which membrane contact between adjacent cells induces outgrowth of TNTs as these cells move apart (74, 75). Even though we did not investigate these mechanisms directly, both models could plausibly explain nanotube formation not only between cells of *D. discoideum* but also among different amoebal species (Figs. 1 and S1A and B). Dynamic actin remodeling and exploratory behavior in motile amoebae are well-documented phenomena (76–79). This might generate protrusions that contact nearby cells and initiate TNT formation. Additionally, transient physical contacts between migrating cells could, in principle, give rise to persistent membrane bridges as the cells move in different directions (17, 74). We could also show that TNT formation occurred de novo and is not the result of incomplete cytokinesis. Moreover, the length of amoebal TNTs, detection of gondolas, a bona fide feature of TNTs, and the formation of both intraspecific and interspecific nanotubes make amoebal TNTs different from other protrusions, including intercellular bridges (10, 11, 46, 80).

TNT formation across multiple forms of life is characterized by the presence of membrane continuity, further transforming TNTs into tubular highways for intercellular transport (10, 81). We elucidated this feature in amoebal TNTs by demonstrating the transport of various cellular cargos and the occurrence of gondolas (Figs. 2, 6C, S5, S4C and D). Another analogy to mammalian TNTs is that amoebal TNTs facilitate the transport of organelles, including mitochondria and actin aggregates, during various stress conditions (82, 83). The co-culture studies involving labeled and unlabeled *Dictyostelium* cells highlight this observation during cytoskeletal stress (Fig. 6). These findings indicate that nanotubes function as essential conduits for the transfer of crucial cellular components to balance out the stress factors, further highlighting the evolutionary significance of TNT-mediated communication. The ability to form nanotubes by amoebae is also indicative of the conserved nature of nanotube biogenesis across various forms of life, bridging prokaryotic and eukaryotic features associated with TNT formation.

Cytoskeletal stress has been shown to elicit variable responses across various eukaryotic systems (56, 58, 84, 85). In budding yeast, latrunculin-mediated disruption of the actin cytoskeleton activates multiple stress-response pathways by inducing various stress markers such as Caf5p efflux pump and Shp1 protein (58, 59, 86). In mammalian systems, the upregulation of TNTs during lat B-induced cytoskeletal stress has similarly been proposed to function as a mechanism to “alarm” neighboring cells within the tissue microenvironment (57). Notably, actin-independent TNTs in both prokaryotic and eukaryotic cells can also be stabilized through other structural elements, including (i) pure phospholipid systems, including anisotropic raft elements (AREs; composed of lipids and proteins) in mammalian cells (52, 55, 73) and (ii) other cytoskeletal elements such as microtubules (87, 88). We observe the localization of microtubules inside TNTs during actin cytoskeleton disruption (Fig. 4). Taken together, these observations suggest that TNT biogenesis under actin-disrupting conditions in amoebae could represent a stress response mechanism that facilitates communication with neighboring cells to balance the cellular dysfunction. However, this hypothesis requires further experimental validation.

One of the key events in eukaryotic evolution, yet still poorly understood, is the transition to multicellularity (89). Along with cell adhesion and differentiation, intercellular communication is also considered a predominant trait driving this transition (90). Multicellularity is not a monophyletic event; rather, it has emerged independently in several early eukaryotic lineages, including protists such as Amoebozoa, where it appears in the form of aggregative multicellularity (41, 91, 92). This involves the aggregation of individual cells into a multicellular sporulation stage, essential for efficient spore dispersal (93). *Myxococcus xanthus*, a soil-dwelling bacterium, also exhibits aggregative multicellularity similar to amoebae, and serves as a prokaryotic model for studying the evolutionary transition to multicellularity (94). Interestingly, TNT-like structures have also been identified in *M. xanthus*, enabling direct connections between individual cells (95). Taken together, the formation of nanotubes among diverse soil-dwelling organisms that exhibit early forms of multicellular behavior strongly suggests the potential role for these nanotubes in the emergence of multicellularity. In natural environments, these nanotubular connections can significantly increase the chances of survival and connectivity among the cells. The emergence of such supracellular assemblies before the evolution of metazoan-type multicellularity may represent a necessary adaptation of early life forms to balance various stress conditions. Hence, understanding the foundations of multicellularity starts with a clear understanding of unicellular mechanisms, including contact-based intercellular communication in early/lower eukaryotes.

**Figure 7:**
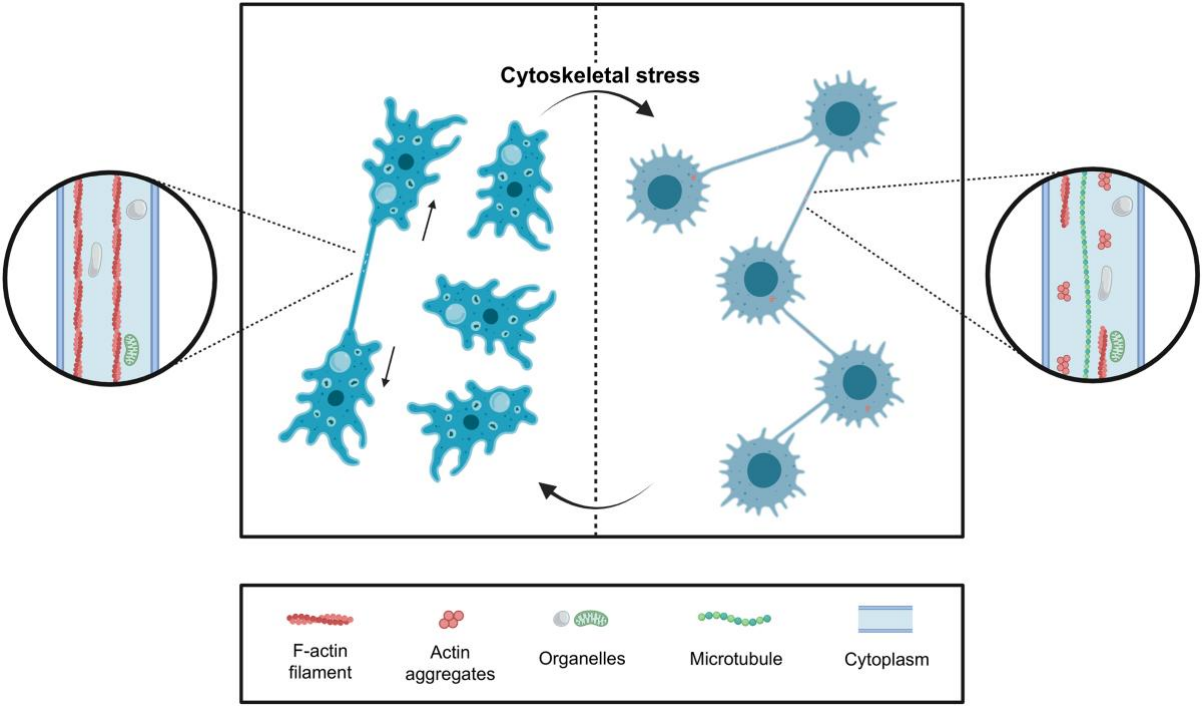
A schematic representation of TNT formation in amoeba discussed in this study. Amoebae can form F-actin-rich nanotubes that establish a direct connection between the cells. This facilitates the transport of various cellular cargoes, such as organelles, through the nanotube structure. During cytoskeletal stress, remodeling of cytoskeletal elements can occur, enabling TNTs to mediate the transfer of cellular cargo. The illustration was created with BioRender.com.

Overall, our study demonstrates the formation of TNTs in amoebae, a nonmammalian model system. The transport of cargo through TNTs provides a functional significance for the biogenesis of TNTs in amoebae. Our study provides the first experimental evidence of functional TNTs in unicellular eukaryotes, beyond the metazoan phyla. The differences in the structural components of nanotubes under different conditions probably highlight the distinct functions of nanotubes in amoebae (Fig. 7). Notably, the detection of TNTs among different strains of *Dictyostelium* and in other amoebal species shows that TNT biogenesis plays a crucial role in intercellular communication, which occurs across multiple clades of amoebae. Our study acts as a valuable framework for expanding the concept of TNT-based supercellularity from metazoan systems to early unicellular eukaryotes, which often exist within polymicrobial communities—offering new perspectives into microbial communication and pathogenesis.

### Limitations of this study and outlook

Investigating TNTs in amoebae presented significant challenges. Amoebae, being fast-moving cells, and the TNTs formed would sever easily as the connected cells move rapidly away from each other, changing the whole morphology of the cells and the TNTs (Fig. S3A and B). TNT structures were highly susceptible to fixation protocols; hence, live-cell imaging was performed throughout the study. Also, live amoebae cells can internalize and exocytose cell staining dyes as vesicles, as part of detoxification, making live-cell staining a difficult process compared to fluorescent reporter expression (96–98). Additional work is required to understand the underlying mechanisms behind the upregulation of TNTs during cytoskeletal perturbations and the interplay between actin and microtubule cytoskeletal elements during TNT formation. Hence, further characterization of amoebal TNTs may provide valuable insights to boost our understanding of the concept of supercellularity outside the metazoan system.

## Materials and methods

### Amoebae culturing

#### Dictyostelium discoideum

*Dictyostelium discoideum* AX2 strain was grown in HL5 medium supplemented with 1% (w/v) glucose (Formedium). *Dictyostelium discoideum* strain AX3 VatMpr/[act15]:vatM:GFP (hereafter *D. discoideum* VGA4; dictyBase accession no. DBS0237042) strain was grown in HL5 medium supplemented with 1% (w/v) glucose and 25 μg mL^−1^ geneticin (G418, InvivoGen). Amoebae strains were grown at 22 °C in cell culture dishes (Sarstedt).

The *D. discoideum* VGA4 strain was used for the extrachromosomal expression of *VatM* gene coupled to GFP (49). The *VatM* gene (encoding for the transmembrane subunit of the V-ATPase enzyme) is used as a reporter for endomembrane organelles in this study (41, 48, 49).

#### *Polysphondylium pallidum* and *P. violaceum*

*Polysphondylium violaceum* Brefeld (ATCC accession no. 34156) and *P. pallidum* PN500 (dictyBase accession no. DBS0302501) were grown on SM/5 agar plates supplemented with *Klebsiella aerogenes* (*Ka*), a typical food bacterium of amoebae. For imaging, the cells were removed from the agar plate by scraping and resuspended in 1× Sörensen's (Sor) buffer (pH 6.0; 2 g L^−1^ KH_2_PO_4_ (Carl Roth), 0.29 g L^−1^ Na_2_HPO_4_ (Carl Roth).

Before an experiment, all the strains were harvested by centrifugation (500 *× g* for 5 min) and washed twice in 1× Sor buffer, unless mentioned otherwise.

### Plasmids and electroporation

#### Actin labeling

The LifeAct peptide was used as a reporter for F-actin filaments (99). Extrachromosomal expression of LifeAct coupled to GFP or RFP was achieved by using the plasmids, pPI138 (addgene #113229) and pDM304, respectively. Using the safe haven knock-in approach, stable expression of LifeAct coupled to mCherry was achieved by using the plasmid pPI226 (addgene #113228) (100).

#### Microtubule labeling

Alpha-tubulin, a well-characterized protein that constitutes microtubules in various organisms, was used as a reporter for microtubules in *Dictyostelium* (101). Extrachromosomal expression of α-tubulin coupled to mRuby3 was achieved by using the plasmid, pDYU1B/mRuby3–α-tubulin (addgene #154302) (102).

#### Electroporation

Axenic cells of *D. discoideum* were grown in HL5 along with the respective antibiotics and were prepared as mentioned above. The cell density was adjusted to 1 × 10^6^ cells mL^−1^ and was resuspended in 30 μL of resuspension buffer (Invitrogen). After the addition of plasmids (1 μg per 10 μL of washed cells), electroporation was performed using the Neon Transfection System (Invitrogen) with three square pulses of 1.8 kV, 10 ms each (29). The plasmids pPI138 and pDM304 were transfected into the *D. discoideum* AX2 strain. *Dictyostelium discoideum* VGA4 cells were transfected with either pPI226 or pDYU1B/mRuby3–α-tubulin for multicolor labeling of actin filaments, microtubules, and endomembrane organelles. *Dictyostelium discoideum* AX2 cells were transfected with the plasmids pPI138 and pDYU1B/mRuby3–α-tubulin for dual-color labeling of actin filaments and microtubules.

After electroporation, *D. discoideum* cells were allowed to recover in HL5 medium for 24 h. For selection, the following antibiotics were used: 25 μg mL^−1^ G418 (extrachromosomal LAG and LAR expression), 50 μg mL^−1^ Hygromycin B (safe haven LAC expression; InvivoGen), and 20 μg mL^−1^ Blasticidin (extrachromosomal mRuby3–α-tubulin expression; InvivoGen). The selection was carried out for 3–5 days.

### Drug treatments

#### Hydroxyurea treatment

To demonstrate that TNTs are not remnants of incomplete cytokinesis, amoebae cells were cultured in the presence or absence of hydroxyurea (HU). In *Dictyostelium*, HU induces cell-cycle arrest at the G1/S phase of cell division (103). *Dictyostelium discoideum* cells expressing LifeAct-RFP (hereafter *D. discoideum* LAR) were grown for 20 h in the presence or absence of 2 mM HU (Thermo Fisher Scientific) (103). After the treatment, the cells were washed twice with 1× Sor buffer. Following the washing step, ca. 3 × 10^5^ of *D. discoideum* LAR cells were seeded in one well of a 4-well µ-Slide containing 400 μL of HL5 medium and allowed to recover for 45 min to 1 h before imaging.

#### Latrunculin and cytochalasin treatment

To understand the influence of actin depolymerization on TNT formation, amoebae cells were treated with latrunculin A (lat A), latrunculin B (lat B), or cytochalasin D (cyt D). Amoebae cells were prepared as mentioned above. For monoculture, ca. 3 × 10^5^ of *D. discoideum* LifeAct-GFP (hereafter *D. discoideum* LAG) cells were seeded in one well of a 4-well µ-Slide containing 400 μL of HL5 medium. To inhibit the actin polymerization, 5 μM lat A (Sigma−Aldrich/Merck), 10 μM lat B (Sigma−Aldrich/Merck), or 50 μM cyt D (Sigma−Aldrich/Merck) were used (60, 61). The equivalent volume of DMSO (vehicle control; Carl Roth) was added to the respective well. The drug treatments were done for 20 min or 1 h at 22 °C. For the rescue, the media containing lat B was removed from the respective well and rinsed twice in 1× Sor buffer. Fresh HL5 media was added to the wells, and the cells were allowed to recover for 30 min prior to imaging.

#### Latrunculin and nocodazole cotreatment

To understand the influence of actin and microtubule depolymerization on TNT formation, amoebae cells were treated with lat B or co-treated lat B and nocodazole (noco). Amoebae cells were prepared as mentioned above. For monoculture, ca. 3 × 10^5^ of *D. discoideum* LifeAct-GFP mRuby3–α-tubulin (hereafter *D. discoideum* LAG-RT) cells were seeded in one well of a 4-well µ-Slide containing 400 μL of HL5 medium. To inhibit the actin and microtubule polymerization, 10 μM lat B and 66 μM noco (61) were used. The treatment was done for 1 h at 22 °C.

### Fluorescence microscopy

#### Imaging of TNTs and organelle transport

*Dictyostelium discoideum* VGA4 cells expressing either LAC (hereafter *D. discoideum* VGA4-LAC) or mRuby3–α-tubulin (hereafter *D. discoideum* VGA4-RT) fusion proteins were used for imaging TNTs and the transport of cargo between the TNT-connected cells. ca. 3 × 10^5^ of *D. discoideum* VGA4-LAC or VGA4-RT cells were seeded in one well of a 4-well µ-Slide (ibidi) containing 400 μL of HL5 medium. The µ-Slide was incubated at 22 °C for 30 min before imaging.

The length of TNTs was determined by measuring the distance from the base of the protrusion of one cell to the point where the TNT joins with the plasma membrane of the other cell (Fig. 1B and C insets) (104). The width was calculated by measuring the diameter at least three different points within individual TNTs.

#### Plasma membrane staining

Wheat germ agglutinin (WGA) staining (WGA Alexa Fluor 647 conjugate, Thermo Fisher Scientific) was used for the live staining of the plasma membrane as per the manufacturer's instructions. The cells were treated with 20 μg mL^−1^ of WGA Alexa Fluor 647 conjugate for 10 min prior to imaging (10, 105). After incubation, the media containing the WGA conjugate was removed and rinsed three times with 1× Sor buffer. Fresh HL5 media was added to the wells and immediately used for imaging to avoid interference of dye internalization.

#### Mitochondria and peroxisome staining

BioTracker 405 Blue or BioTracker 488 Green Mitochondria Dye (Sigma−Aldrich/Merck) and Peroxi_SPY™555 (Spirochrome) was used for the live staining of mitochondria and peroxisomes, respectively, as per the manufacturer's instructions. For mitochondrial staining, the cells were treated with 300 nM of either BioTracker 405 Blue or BioTracker 488 for 15 min prior to imaging. For peroxisomal staining, the cells were treated with 5 μM of the dye for 1 h prior to imaging.

#### Co-culture setup

The co-culture between *P. violaceum* and *D. discoideum* VGA4-LAC or LAG strains was set up in a 4-well µ-slide in a 1:1 ratio, resulting in a total cell density of 3 × 10^5^ amoebae cells per well. Since *P. violaceum* is not adapted to be an axenic strain, *Ka* was also present in the co-culture.

The other axenic co-cultures: (i) *D. discoideum* VGA4 and *D. discoideum* LAR and (ii) unlabeled *D. discoideum* AX2 and *D. discoideum* VGA4-LAC were also made in a similar manner in a 4-well µ-slide. The cells were treated with 10 μM lat B for 1 h prior to imaging.

#### Imaging parameters

Live-cell imaging was performed using a spinning disk confocal laser scanning microscope (AxioObserver.Z1/7, Carl Zeiss) equipped with a 63×/1.2 NA LD LCI Plan-Apochromat oil-immersion Imm Corr DIC (Carl Zeiss) and 100×/1.40 NA Plan-Apochromat oil-immersion DIC M27 objective lens (Carl Zeiss). Images were captured using the diode lasers: 405 nm (50 mW), 488 nm (50 mW), 561 nm (50 mW), and 638 nm (75 mW) with the corresponding emission bandpass (BP) filters: BP 450/50, 485/30, BP 600/50, and BP 629/62. The images were captured as z-stacks with 15–20 optical sections (0.50 and 0.20 µm per section for 63× and 100× magnifications, respectively), 2 × 2-pixel binning, and 2× gain. Brightness and contrast were adjusted accordingly in some images, only to increase the visualization of nanotubular connections.

Images of long TNTs were captured at 63× magnification using the tile scan mode coupled with z-stack acquisition. The images were captured as 2 × 2 tile z-stacks, followed by tile stitching using ZEN blue software to generate high-resolution tiled images of TNTs. The movement of organelles inside TNTs was captured as time-lapse images at 63× magnification with 4 × 4-pixel binning and 3× gain.

### Image analysis

Images were processed using ZEN 2.6 (Blue edition, Carl Zeiss) imaging software. The z-stack images were also subjected to deblurring (strength-0.4, blur radius-50, and sharpness-0.1) in the ZEN Blue software. Orthogonal projection was also obtained using the same software. The measurements of TNTs, depth coding, and 2D maximum projections of z-stacks were obtained using ImageJ 1.54p (Fiji) software (106).

### Quantification of TNTs

The amoebae cells were counted using the CellProfiler (107) software (version 4.2.1). The brightfield images were imported into a pipeline having two modules: (i) RescaleIntensity and (ii) RunCellpose (Cellpose 1.0.2) modules to segment and count the cells. The pretrained “cyto2” detection network with automatic detection and a flow threshold of 0.4 was implemented.

Both the number of TNTs and TNT-connected cells were counted following these criteria: F-actin-positive, thin, membranous structures are present connecting two or more cells, and these connections are at least partially nonadherent to the substratum (68, 108). Cells that are not in contact with each other were considered negative (no TNT connection). Each cell connected by a TNT to another cell was counted as positive. This was further represented as the percentage of cells with TNT, calculated according to the formula:


$$\begin{array}{rl} \mathrm{Percentage}\;\mathrm{of}\;\mathrm{cells}\;\mathrm{with}\;\mathrm{TNT}= & \left(\frac{\mathrm{Total}\;\mathrm{no}.\;\mathrm{of}\;\mathrm{cells}\;\mathrm{with}\;\mathrm{TNTs}}{\mathrm{Total}\;\mathrm{no}.\;\mathrm{of}\;\mathrm{amoebae}\;\mathrm{counted}}\right)\\ & \times 100. \end{array}$$

The percentage of TNTs (relative to the total number of cells) was calculated according to the formula:


$$\mathrm{Percentage}\;\mathrm{of}\;\mathrm{TNT}=\left(\frac{\mathrm{Total}\;\mathrm{no}.\;\mathrm{of}\;\mathrm{TNTs}}{\mathrm{Total}\;\mathrm{no}.\;\mathrm{of}\;\mathrm{amoebae}\;\mathrm{counted}}\right)\times 100.$$

For each experiment, a total of 10 images from each parameter were used for the analyses. The percentages were represented as bar plots with individual values for each experiment. The experiments were performed in three independent replicates.

### Statistical analysis

Prism 10 (GraphPad) software was used to perform statistical analysis and prepare the graphs. All experiments were performed with at least three biological replicates. The statistical analyses were performed using a nonparametric Kruskal–Wallis test followed by Dunn's post hoc test for multiple comparisons. To define significance, a *P* value of less than 0.05 was used. Asterisks (*) are used in the figures to indicate different significance levels. Data shown represent mean ± standard error.

## Supplementary Material

pgag238_Supplementary_Data

## Data Availability

All data supporting the findings are available in the main text or the supplementary materials. The strains generated by this study are available from the corresponding author upon request.
