# Supplementary material for: Nanotubes enable intercellular communication in early-branching eukaryotes
Source: PNAS Nexus. 2026 Jul 28;5(7):pgag238. doi: 10.1093/pnasnexus/pgag238 (PMC13410376; doi:10.1093/pnasnexus/pgag238)
Supplement: pgag238_Supplementary_Data [file pgag238_supplementary_data.pdf]

Supporting information for

Nanotubes enable intercellular communication in early-branching eukaryotes

Harikumar R. Suma[1,2#], Robert R. Kay[3], Sandeep M. Eswarappa[4], and Pierre Stallforth[1,5,*]

[1]Department of Paleobiotechnology, Leibniz Institute for Natural Product Research and Infection Biology – Leibniz-HKI, Beutenbergstrasse 11a, 07745 Jena, Germany

[2]Cluster of Excellence Balance of the Microverse, Friedrich Schiller University, Fürstengraben 1, 07743 Jena, Germany

[3]MRC Laboratory of Molecular Biology, Cambridge CB2 0QH, UK

[4]Department of Biochemistry, Indian Institute of Science, Bengaluru, 560012 Karnataka, India

[5]Faculty of Chemistry and Earth Sciences, Friedrich Schiller University (FSU), Humboldtstrasse 10, 07743 Jena, Germany

#Current affiliation: Department of Biotechnology and Biomedicine, Technical University of Denmark, Søltofts Plads Bldg. 221, 2800 Kgs. Lyngby, Denmark

[*]Correspondence: pierre.stallforth@leibniz-hki.de (P.S.)

This PDF file includes:

Figure S1 to S6

Figure. S1. TNTs in amoebae. (**A – B**), Formation of interspecific TNT-like protrusions between different species of amoebae. **a**, Brightfield images of co-culture between *D. discoideum* VGA4-LAC and *P. violaceum* (*P. vio*) cells. (**B**), Brightfield images of co-culture between *D. discoideum* AX2 LAG (AX2) and *P. violaceum* (*P. vio*) cells. TNT-like protrusions were identified between both species of amoebae. The absence of fluorescence in these structures indicates that the nanotubes are formed by protrusions from the unlabeled *P. violaceum* strain. Yellow arrowheads indicate the interspecific nanotubes formed between *D. discoideum* and *P. violaceum* cells. White arrowheads indicate the intraspecific nanotubes between *P. violaceum* cells. Scale bars, 10 µm. (**C**), Tiled and stitched image of a TNT connecting two *D. discoideum* VGA4-LAC cells. Blue arrowheads indicate organelles, and orange arrowheads indicate the actin-positive TNT. The entire length of this TNT is *ca.* 450 µm. Scale bar, 20 µm. (**D**) Quantification of TNTs formed between *D. discoideum* AX2 LAR cells in the presence or absence of hydroxyurea. Statistical significance was determined using the Mann-Whitney test (n=10 images). The differences in the number of TNTs between treated and untreated cells were not significant (symbols: ns, not significant). Hence, amoebal TNTs are not remnants of incomplete cytokinesis.

Figure. S2. Z-profile of amoebal TNTs.

(**A**) Serial z-stack images of an amoebal TNT (see Fig 1B), acquired with a step size of 0.8 µm. Scale bar, 10 µm (**B**) Depth-coded projection of (A). Colors represent the z-depth (blue: bottom and red: top). (**C**) Orthogonal view of Fig 2A showing the X-Y and XZ planes. Orange arrowheads indicate F-actin rich TNTs, and blue arrowheads indicate endomembrane organelles. These images show that amoebal TNTs do not adhere to the substratum. Scale bar, 10 µm.

Figure. S3. TNTs are dynamic structures and vulnerable to amoebal locomotion.

(**A**) Time-lapse imaging shows two *D. discoideum* VGA4-LAC cells connected by a TNT. As soon as live-cell imaging is started, retraction of the nanotube can be observed. Time format is minutes:second. Scale bar, 10 µm (top panel) and 5 µm (bottom panel). The images were captured as time-lapse z-stacks with 2 × 2-pixel binning, and 2x gain. (**B**) Time-lapse imaging shows the movement of a *D. discoideum* VGA4-LAC amoeba beneath a TNT connecting two other *D. discoideum* VGA4-LAC cells. Images shown are overlay of actin (orange) and brightfield channels. These observations highlight that TNTs are not cleaved by amoebic midwifery (1, 2) as they are suspended above the substrate. However, they are affected by the photo sensitive behavior of amoebae (3, 4). They can retract, extend, or shorten according to the movement of amoebae. The images were captured as time-lapse z-stacks with 4 × 4-pixel binning, and 3x gain. White arrow indicates the amoebae moving beneath the nanotube. Time format is minutes:second. Scale bar, 10 µm.

Figure. S4. Absence of microtubules and occurrence of gondolas on amoebal TNTs. (**A**) Microtubule localization is absent inside TNTs that support cargo transfer in *D. discoideum* VGA4-LAC cells. Blue arrowhead indicates organelles inside the TNT. Scale bar, 10 μm. (**B**) Microtubule localization is absent inside TNTs that form between *discoideum* LAG-RT cells. Blue arrowhead indicates actin-positive TNT, and green arrowhead indicates the absence of microtubules. Scale bar, 10 μm. (**C – D**) Gondola structures were identified on TNTs formed between *D. discoideum* VGA4-LAC cells. In the bottom panel. Signals for organelles were detected inside the gondola, indicating that these distensions act as carriers for transporting the cellular cargo. Orange arrowheads indicate gondolas on actin-positive TNTs; blue arrowheads indicate organelles inside the gondola; yellow arrowheads indicate the gondolas in brightfield, and white arrowheads indicate the gondolas in the overlay of all channels. Scale bars, 10 μm. (**C**) Presence of a gondola-like structure on a TNT formed by *D. discoideum* LAG cells. Blue arrowheads indicate gondolas on actin-positive TNTs; yellow and white arrowheads indicate the gondola in brightfield and the overlay, respectively. Scale bars, 10 μm.

Figure. S5. Movement of cargo inside amoebal TNTs.

Time-lapse imaging shows the movement of endomembrane organelles (blue) between two *D. discoideum* VGA4-LAC cells connected by an actin-rich TNT (orange). Individual organelle movement is indicated by magenta, white, and green arrowheads. Time format is minutes:second. The images were captured as time-lapse z-stacks with 4 × 4-pixel binning, and 3x gain. Scale bar, 10 μm (top panel) and 5 μm (bottom panel).

Figure. S6. Properties of cytoskeletal stress-induced TNTS.

(**A**) WGA staining of *D. discoideum* LAG cells following lat B treatment shows that membrane continuity is still maintained in TNTs during disruption of the actin cytoskeleton. Blue and white arrowheads indicate TNT with actin aggregates, and the WGA signal on the nanotube, respectively. Scale bar, 10 μm. (**B**) An incomplete nanotube protrusion with actin aggregates extending towards the neighboring cell during lat B treatment. Blue arrowheads indicate the protrusion with actin aggregates; yellow and white arrowheads indicate protrusion in brightfield and the overlay, respectively. Scale bar, 10 μm. (**C**) Effect of lat A treatment (5 μM) on TNT formation in amoebae. During both treatments (20 min: top panel and 1 h: bottom panel) cells lose their amoeboid morphology and start to round up. In both cases, TNTs were detected, suggesting that both latrunculin A and B treatments can induce TNT formation. Scale bar, 10 μm.

References

1. A. Nagasaki, T. Q. P. Uyeda, Chemotaxis-mediated scission contributes to efficient cytokinesis in *Dictyostelium*. *Cell Motil.* 65, 896–903 (2008).
2. D. Biron, P. Libros, D. Sagi, D. Mirelman, E. Moses, “Midwives” assist dividing amoebae. *Nature* 410, 430–430 (2001).
3. P. R. Fisher, D. P. HÃ¤der, K. L. Williams, Multidirectional phototaxis by *Dictyostelium discoideum* amoebae. *FEMS Microbiol. Lett.* 29, 43–47 (1985).
4. D.-P. Häder, M. Watanabe, M. Furuya, Multiple Photoreceptors in Phototaxis of *Dictyostelium discoideum* Amoebae in *Cell Dynamics*, 1st Ed., M. Tazawa, Ed. (Springer, Vienna, 1988), pp. 155–161.

Figure S1

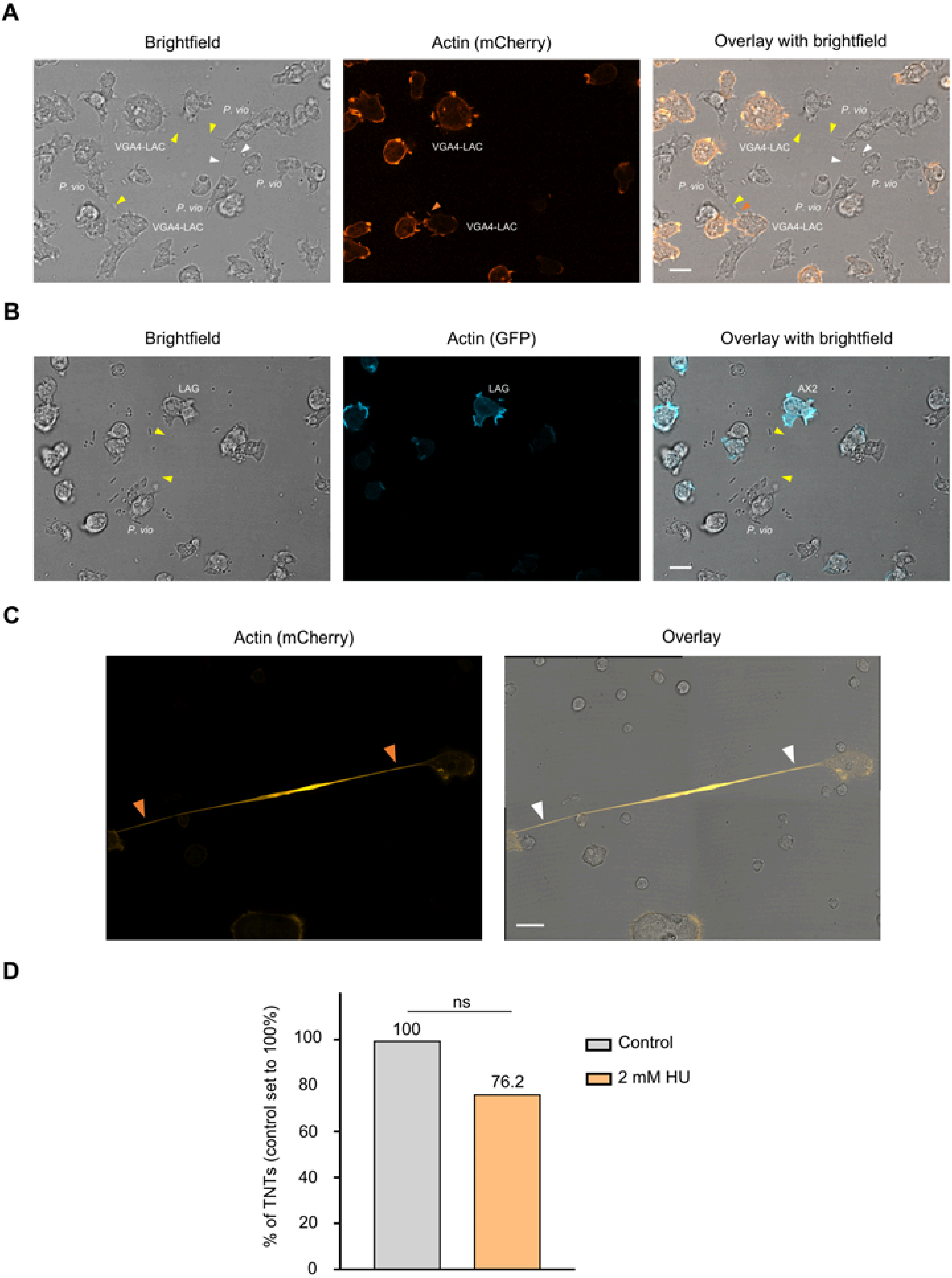
A
Brightfield
Actin (mCherry)
Overlay with brightfield
P. vio
VGA4-LAC
P. vio
P. vio
P. vio
VGA4-LAC
B
Brightfield
Actin (GFP)
Overlay with brightfield
LAG
P. vio
LAG
AX2
C
Actin (mCherry)
Overlay
D
% of TNTs (control set to 100%)
0
20
40
60
80
100
ns
100
76.2
Control
2 mM HU

Figure S2

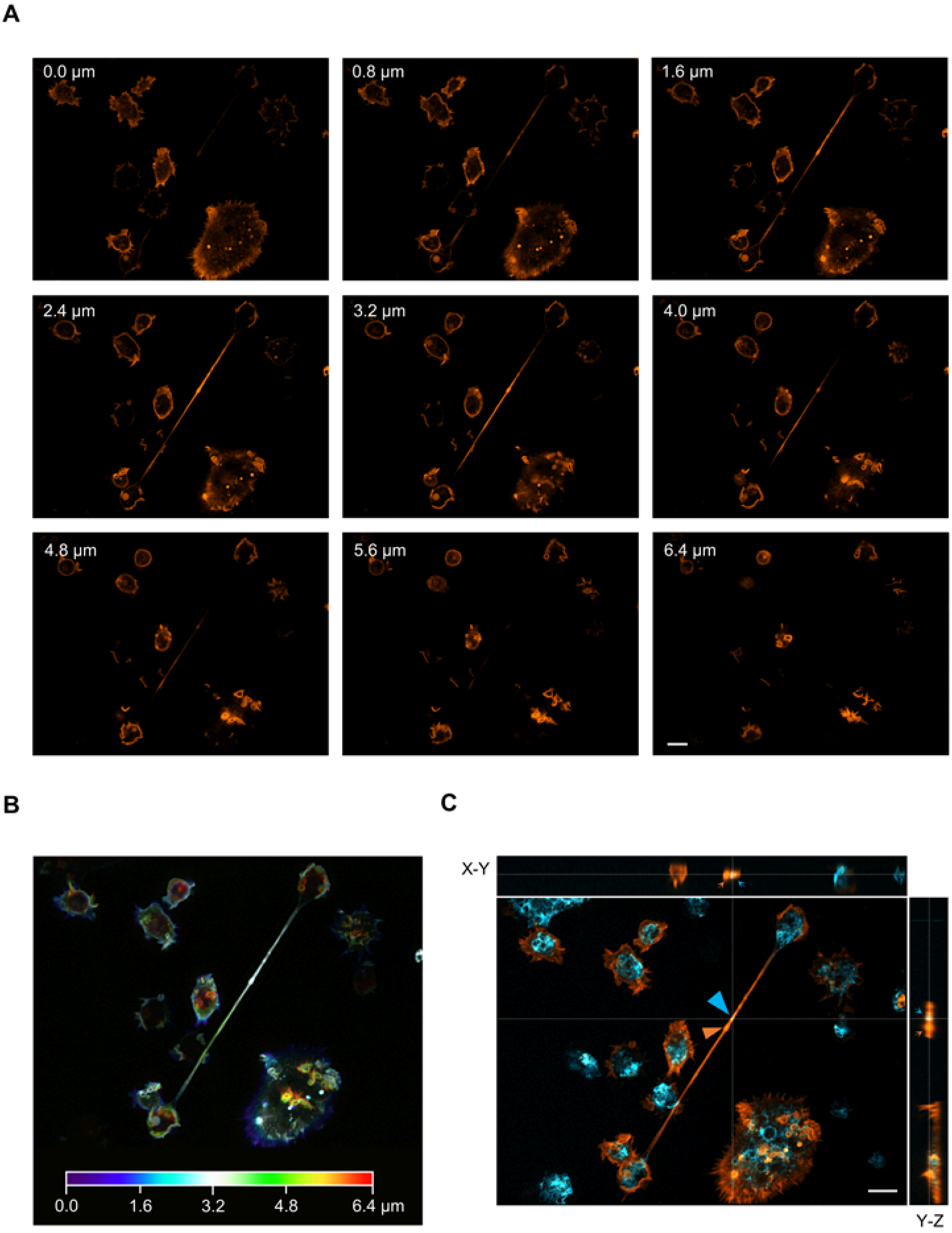
A
0.0 μm
0.8 μm
1.6 μm
2.4 μm
3.2 μm
4.0 μm
4.8 μm
5.6 μm
6.4 μm
B
0.0
1.6
3.2
4.8
6.4 μm
C
X-Y
Y-Z

Figure S3

A

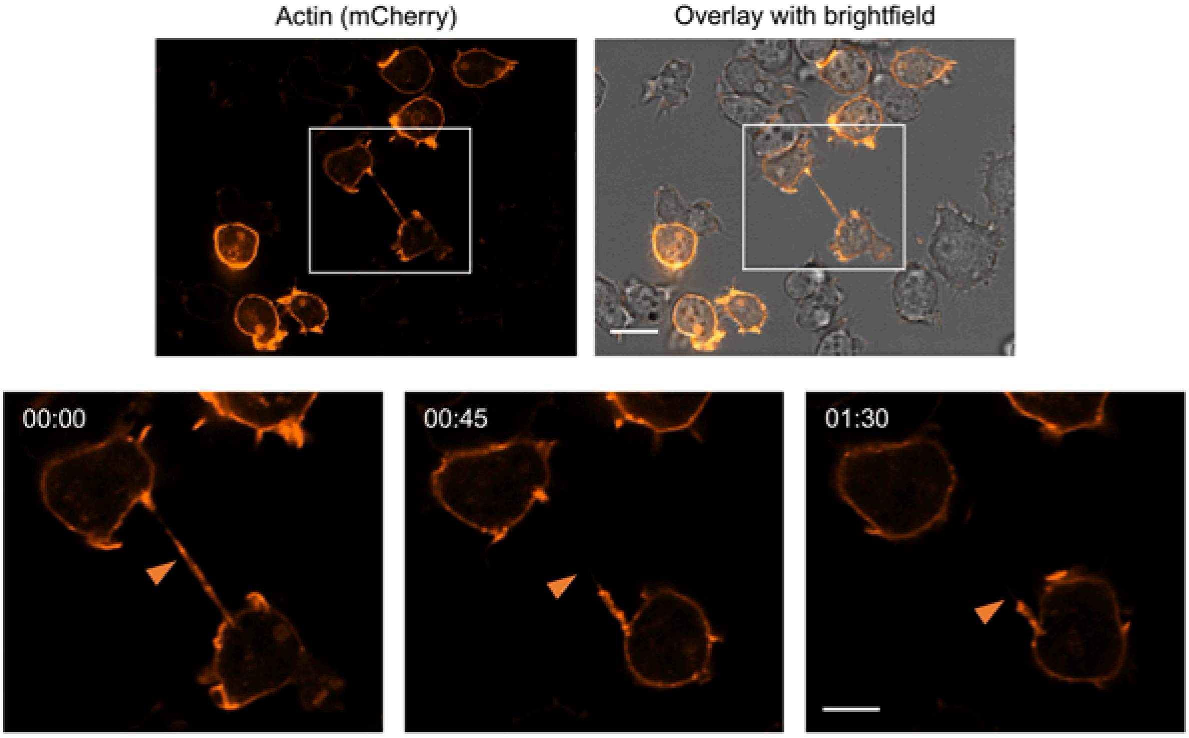
Actin (mCherry)
Overlay with brightfield
00:00
00:45
01:30

B

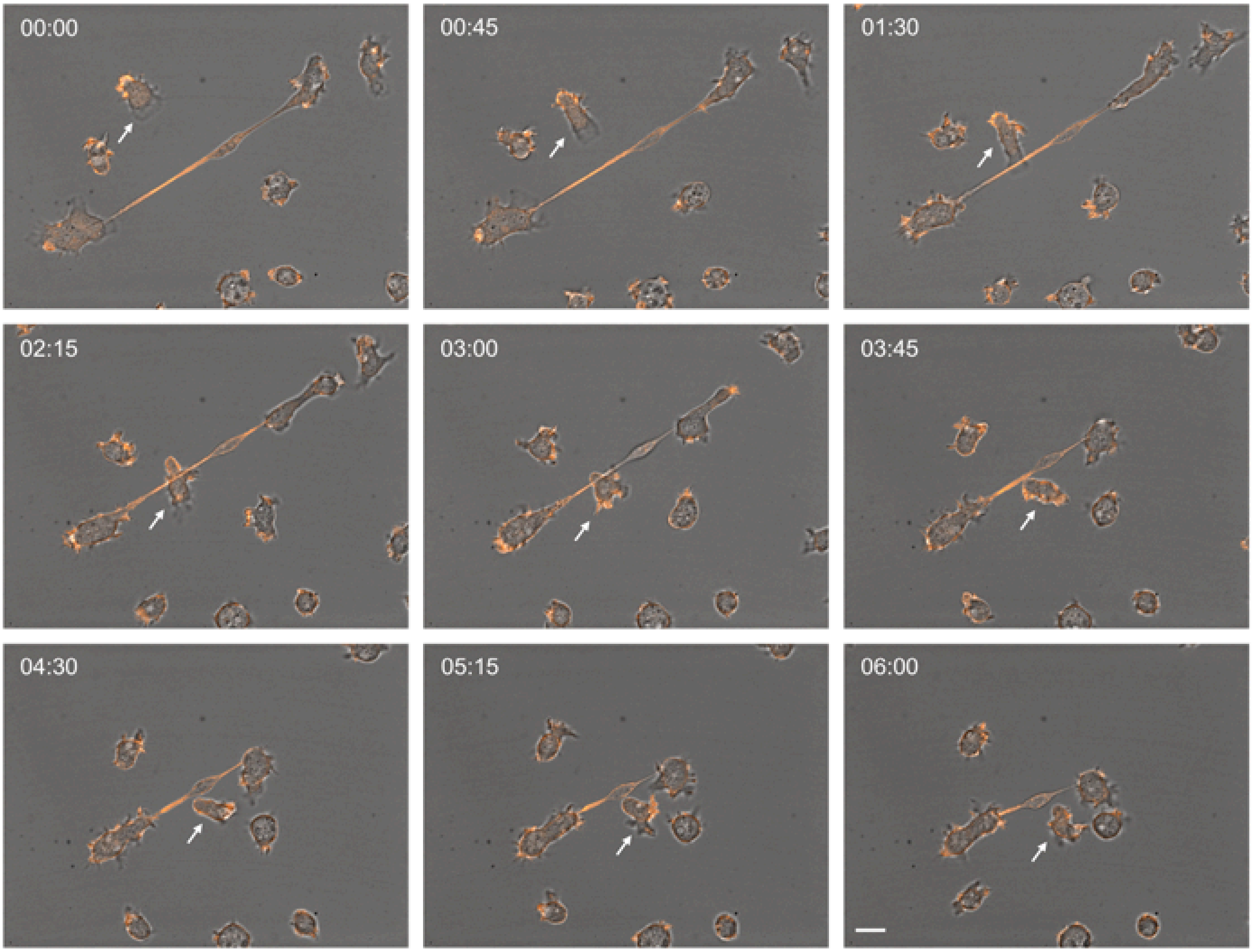
00:00
00:45
01:30
02:15
03:00
03:45
04:30
05:15
06:00

Figure S4

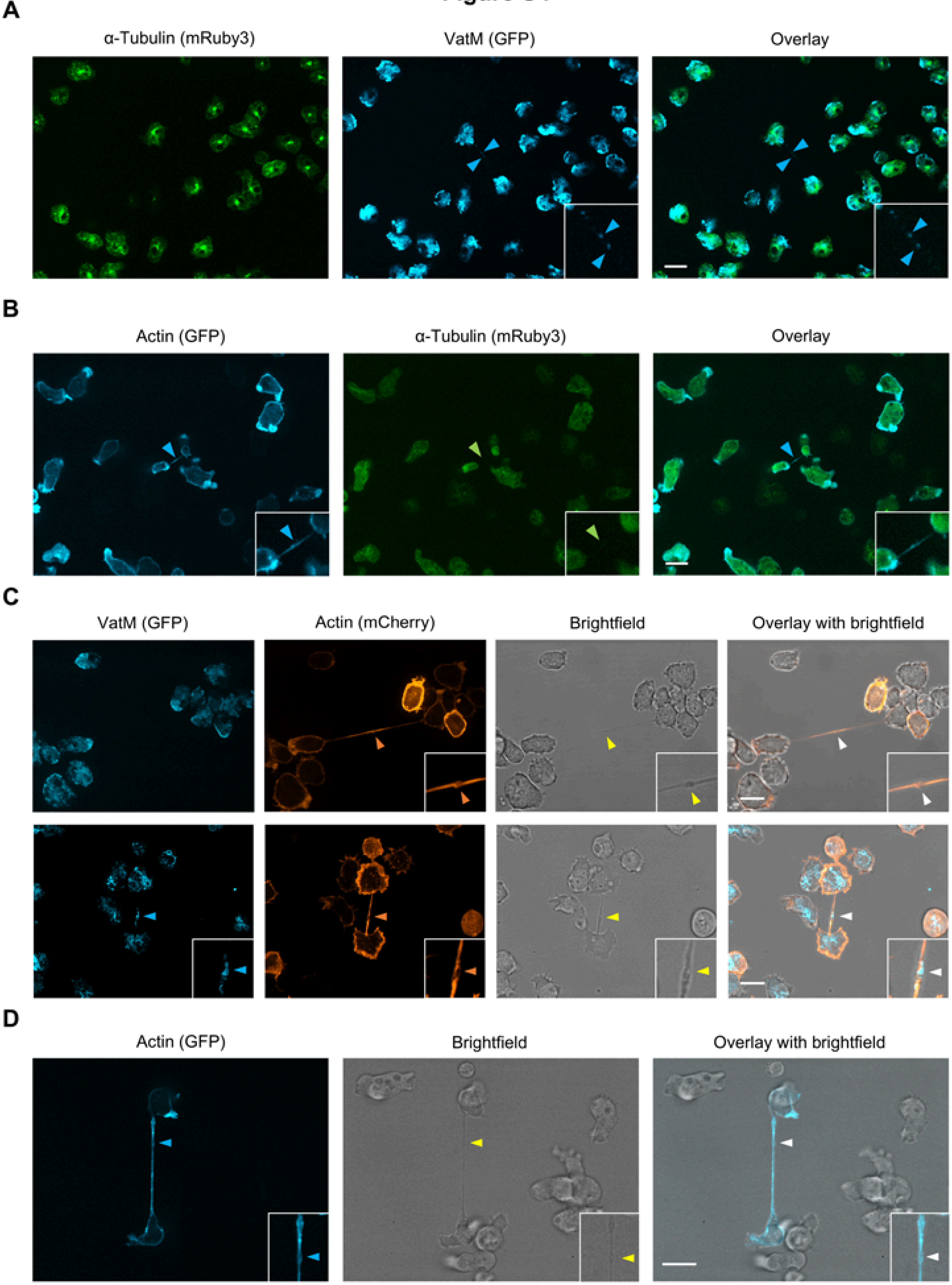
A
α-Tubulin (mRuby3)
VatM (GFP)
Overlay
B
Actin (GFP)
α-Tubulin (mRuby3)
Overlay
C
VatM (GFP)
Actin (mCherry)
Brightfield
Overlay with brightfield
D
Actin (GFP)
Brightfield
Overlay with brightfield

Figure S5

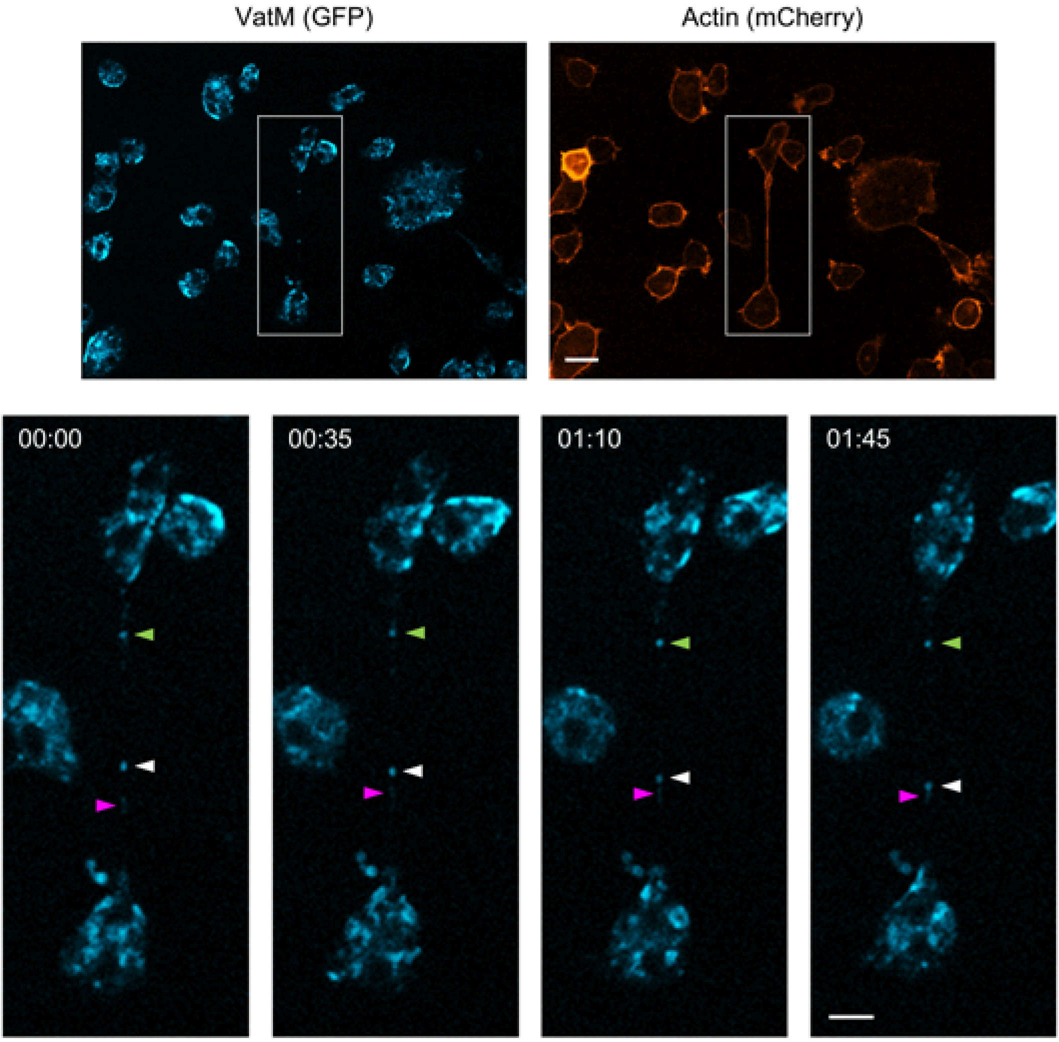

Figure S6

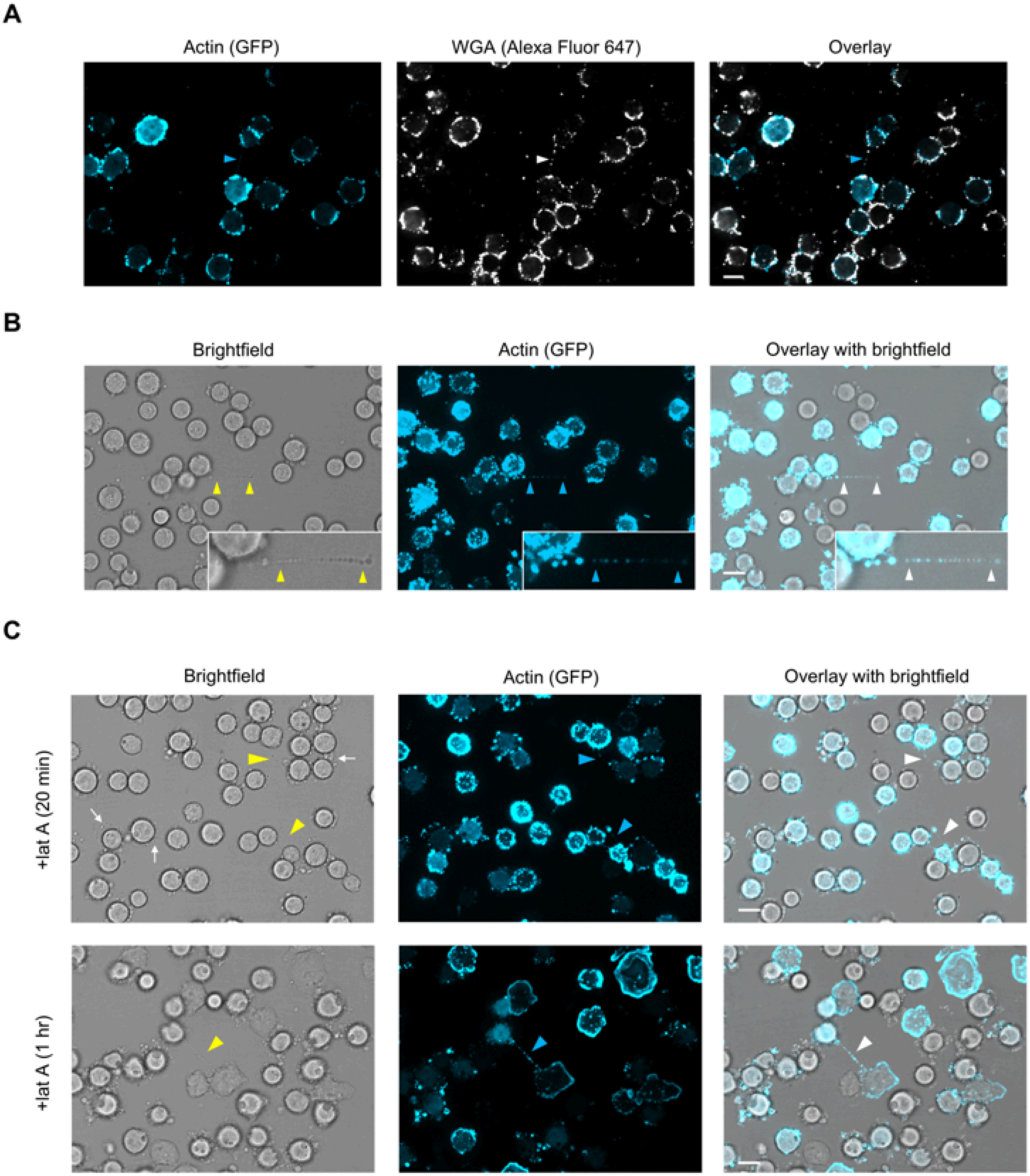
A
Actin (GFP)
WGA (Alexa Fluor 647)
Overlay
B
Brightfield
Actin (GFP)
Overlay with brightfield
C
Brightfield
Actin (GFP)
Overlay with brightfield
+lat A (20 min)
+lat A (1 hr)
